# Use of levothyroxine in the management of hypothyroidism: A historical perspective

**DOI:** 10.3389/fendo.2022.1054983

**Published:** 2022-11-02

**Authors:** George J. Kahaly, Ulrike Gottwald-Hostalek

**Affiliations:** ^1^ Department of Medicine I, Johannes Gutenberg University (JGU) Medical Center, Mainz, Germany; ^2^ Research and Development, Merck Healthcare KGaA, Darmstadt, Germany

**Keywords:** levothyroxine, thyroxine, hypothyroidism, thyroid gland, history of medicine

## Abstract

The thyroid operates within a complex system of homeostatic regulation, where the level of thyrotropin (TSH) influences the rate of secretion of the principal thyroid hormones, thyroxine (T4) and triiodothyronine (T3). The devastating consequences of untreated thyroid dysfunction have been evident for centuries. Indeed, sources from antiquity described goitre and cretinism, two of the clinical sequelae of untreated overt thyroid disease. It was not until the first part of the 19^th^ century that goitre and cretinism were first associated with iodine status; however, the endocrine function of the thyroid was not clearly identified until the early part of the 20^th^ century. Three principal innovations in the 20^th^ century supported the use of levothyroxine (LT4) replacement therapy for the management of hypothyroidism: a practical technique for the synthesis of LT4 suitable to support pharmaceutical use (late 1940s), the discovery that LT4 is converted to the active thyroid hormone, T3, in the peripheral tissues (1970), and the development of robust and sensitive assay methodology for measuring thyroid hormones in the blood (1960 onwards). Synthetic LT4, titrated to bring the level of TSH within a predefined “normal” reference range, is now established as the mainstay of treatment for hypothyroidism, and provides adequate restoration of thyroid hormone function for most people with this condition. Future research will explore further the nuances of the hypothalamic-pituitary-thyroid axis, and the place, if any, for T3 within the management of thyroid dysfunction.

## Introduction – Current management of hypothyroidism

**Figure 1:**
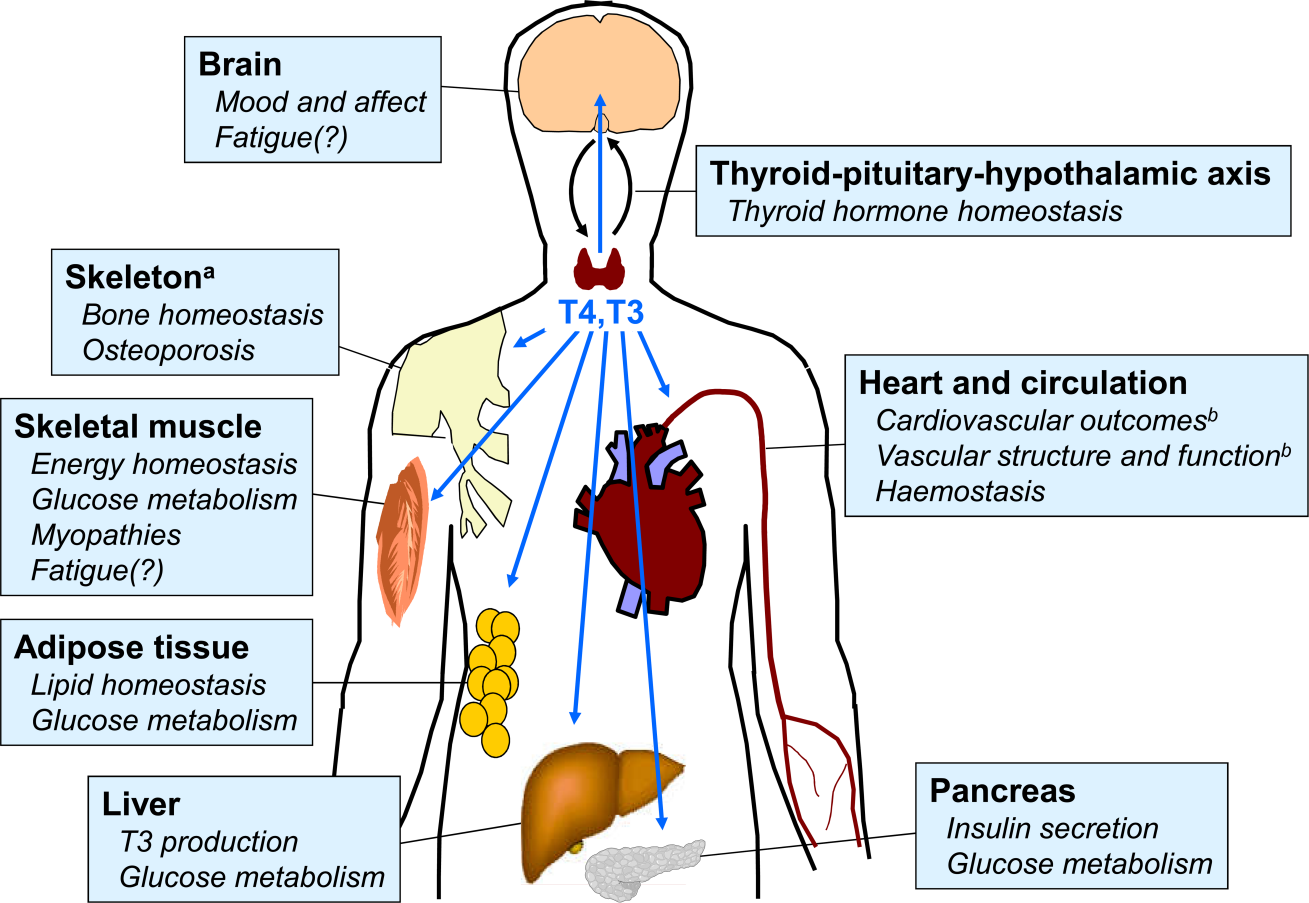
Overview of principal target tissues for thyroid hormones. Areas with question marks are speculative and remain the subject of research. Reproduced from reference 2 under Creative Commons Attribution 4.0 International License (http://creativecommons.org/licenses/by/4.0/). See original source for references.

Thyroid homeostasis occurs through a complex system of overlapping feedback loops (1). Briefly, the secretion of thyrotropin (thyroid stimulating hormone; TSH) from the pituitary stimulates the thyroid gland to secrete two principal hormones: triiodothyronine (T3) and levothyroxine (T4), with T4 accounting for about 80% of the total. These hormones act on almost every organ system in the body. T3 is the active hormone, and T4 from the thyroid gland is converted to T3 by deiodinases within target tissues (**Figure 1**) (2). The levels of T3 and T4 in the circulation feed back to circuits in the hypothalamus that regulate the secretion and activity of thyrotropin releasing hormone, which in turn influences the secretion of TSH. Thus, the overall effects of the thyroid in the body is determined not only by feedback loops between the thyroid and the brain, but also by the activity of deiodinases within the target tissues, among other systems (1).

Hypothyroidism, a state of deficiency of thyroid hormones, is diagnosed mainly according to the circulating level of serum TSH (3–5). When the secretion of thyroid hormones is abnormally low, the pituitary secretes more TSH. Importantly, this relationship is not linear, and a reduction in circulating free T4 (FT4) by half would stimulate an increase in TSH secretion by as much as 100-fold (6). For this reason, the diagnosis of hypothyroidism is based mainly on the level of serum TSH, with levels of other thyroid hormones used to confirm the diagnosis (3–5). A level of serum TSH above an assay-specific reference range of TSH levels (typically around 0.4–4 mIU/L) determined in a population believed to be free of thyroid dysfunction is indicative of the presence of hypothyroidism; “subclinical hypothyroidism” refers to a state where serum TSH is elevated but thyroid hormone levels are normal (7).

The current management of overt hypothyroidism is based firmly on hormone replacement therapy with levothyroxine (LT4) a synthetic form of T4 (3–5). Careful adjustment of the dose of LT4 over time is used to bring TSH back to within its reference range, which provides sufficient restoration of thyroid function for most people with hypothyroidism. The therapeutic use of T3 for people with hypothyroidism remains controversial, and is discussed briefly in the “looking ahead” section at the end of this article.

More than two centuries of research have led us to this point. This article presents a concise overview of the history of the development of LT4 for the management of hypothyroidism.

## Establishing a role for the thyroid gland

### Early observations

It is clear from historical records that the clinical sequelae of hypothyroidism have always been with us. Iodine deficiency is a common cause of an underactive thyroid gland (8), leading to the development of goitre. Hilly or mountainous regions are often low in iodine, as this element has been washed down to lower levels over time. Many sources, reviewed elsewhere (9–11), have noted a high prevalence of goitre in mountainous regions. For example, writings from ancient sources in China include associations between consumption of mountain spring water and goitre, or attempts to treat water with burnt sponge and seaweed that go back as far as 1600 BCE. In Europe, goitres featured prominently in Roman art (12) and in some Renaissance paintings, including ‘A Grotesque head’, by Leonardo da Vinci, and “The Adoration of Shepherds’ by Moretto da Brescia (13–15). The endemic nature of goitre in some regions accounts for the common representation of this condition in ancient art (16). Goitre is also described in near-eastern and Ayurvedic texts from the early centuries BCE (17). Observations of the high prevalence of goitre in many other mountainous regions have been made (9–11), and as recently as 1966 a survey of the prevalence of “Derbyshire neck” appeared in *The Lancet*, referring to an area situated in the southern end of the Pennine hills in the Midlands of England (18).

The early observations, including those pertaining to treatment of goitre with preparations derived from iodine-containing sponge or seaweed, were not based on an understanding of the physiological role of the thyroid gland. The building blocks for our understanding of the importance of the thyroid gland in health and disease were laid in the middle and latter part of the 19^th^ century. Physicians began to describe isolated cases of cretinism, a clinical consequence of untreated severe congenital hypothyroidism. For example, a report of two cases in 1849 described neck swellings in children with cretinism, one confined to an “*Idiot Asylum*” and one referred as an infant, each with apparent severe growth retardation (19). Both died shortly after.

### Limited progress in the 19^th^ century

In 1871, Dr Fagge described “*sporadic cretinism*”, an endemic condition in a village in the southwest of England, which was “*analogous to the cretinism of Alpine countries, and like it, frequently associated with goitre and deaf-mutism*” (20). Public health measures, including better nutrition and encouragement to conduct fewer consanguineous marriages, appeared to resolve that situation. Sir William Withey Gull, describing the decline in mental and physical status of a woman who developed cretinism in adulthood, provided in 1874 what was perhaps the first longitudinal data on the clinical consequences of thyroid deficiency [summarised by Pearce (21)]. William Ord, in 1878, presented more longitudinal data, describing the case of a woman whose mental and physician condition declined severely over a seven-year period due to untreated thyroid disease, leading to her death (22). Interestingly, Dr Ord likened the pathological changes in the presentation of the patient to those described by Sir William Gull, mentioned above. Dr Ord coined the term, “*myxoedema*” at this time, to describe the “*mucus oedema*” displayed by this patient, a term associated with thyroid dysfunction to this day. Soon afterwards, in 1885, Hirsch, described cases of goitre and cretinism in Germany that were far more common on mountainous regions than in iodine-rich coastal regions (23).

There was still no real understanding of the links between goitre, cretinism and the thyroid, despite pointers from the result of experiments on animals subjected to thyroidectomy (and in one case, re-grafting the thyroid to elsewhere in the body) (24, 25). This lack of understanding did not restrain some physicians from excising goitres to relieve pressure on the neck: one surgeon writing in 1883 moved away from performing complete thyroidectomy for this purpose, as it brought on the unwanted outcome of “sporadic cretinism” (26). Dr Ord discussed but largely discounted the possible relationship between the symptoms he described and possible thyroid dysfunction, noting that (referring to Fagge’s work, above) that “*while goitre was more or less associated with endemic cretinism, the thyroid gland was actually absent or atrophied in sporadic cretinism occurring in this country*) (22). The concepts of acquired and congenital hypothyroidism would not be understood until many years later.

### Towards an understanding of thyroid function

Understanding of the likely importance of iodine in the pathogenesis of goitre had been increasing from 1820–1870, including limited trials of administration of iodine preparations for this purpose (27–29) One such attempt in France foundered initially due to problems with iatrogenic thyrotoxicosis due to over treatment, resistance from the established medical profession, and a desire from a number of potential patients to retain their goitres, which provided an exemption from forced conscription into the French army at that time (27). Chatin, in France in 1851 first published the hypothesis that iodine was important here, followed by an observation by Semon (who worked with Ord in London) that myxoedema followed thyroidectomy (30). Successful trials followed of injections of extracts of sheep thyroid for people with myxoedema from 1890 onwards (3). One report showed that a patient with advanced myxoedema responded remarkably to treatment with sheep thyroid extract, and lived for a further 28 years until his death at age 74 years (31). A subsequent review in 1893 of 100 cases of patients with myxoedema and cretinism used phrases such as “*complete transformation*” and “*the patient has ceased to be a patient*” to describe the remarkable efficacy of sheep thyroid extract for these patients (32).

At last, we had not only a firm association between myxoedema and thyroid dysfunction, but also the beginnings of how to treat it. Further progress required further research, however. A pivotal moment in the history of thyroid research was the discovery of “thyro-iodine” a substance containing iodine located within the thyroid gland, by Baumann in 1895 (33). This observation helped to coalesce into a coherent schema the earlier observations of iodine and thyroid function described above. Other researchers confirmed and extended this work, identifying other iodine-containing substances within the thyroid gland [reviewed elsewhere (34)] before Kendall isolated T4 from thyroid extract in 1914 (35). The profound metabolic effects of this newly discovered compound when administered to animals provided the initial basis for our current understanding of the thyroid as an endocrine gland.

## Establishment of LT4 as the standard of care for hypothyroidism

Much useful work had been done in the preceding decades relating to the need for careful dose titration of thyroid extract to preserve an acceptable balance between efficacy and safety, principles that we still follow today (36), although progress in establishing LT4-based therapy was slow in the early part of the 20^th^ century. T4 was not synthesised *de novo* until 1927 (with demonstration that the levoisomer of thyroxine exerts the clinical effects) (37). A synthesis of LT4 suitable for use on a commercial scale for pharmaceutical use did not follow until 1946 (38). This preparation was synthesised as an acid, resulting in low bioavailability, a situation improved by the production of a more soluble sodium salt in 1949 (39).

Meanwhile, thyroid extracts were used for treating myxoedema or hypothyroidism, where the condition was treated at all, as attempts to refine T4 from animal thyroid resulted in a very low yield; for example Kendall found in 1919 that “*Up to the present time about 33 gram of the compound have been separated from 6,550 pounds of fresh [porcine] thyroid material*” (40; NB: 6,550 pounds equates to 2.97 tonnes). The availability of pharmaceutical-grade LT4 from the 1940s did not prevent the widespread use of animal thyroid extracts, which predominated until well into the second half of the 20^th^ century, despite issues such as widely variable (or no) thyroid hormone content and limited shelf life (10, 11).

The discovery of peripheral conversion of T4 to T3 independently of the thyroid in 1970 (40) [now known to be mediated by a family of specific deiodinase enzymes (41)] helped to allay concerns expressed by physicians at the time that monotherapy with LT4 might deplete physiological pools of T3 (10). The development of practical tests for thyroid function was also an important breakthrough. A test for total T4 was first developed in 1960, followed by commercial tests for TSH and T3 [which had been discovered in 1952 (42)] in the mid-1970s. Now, sensitive and specific assays are available to measure T4 or T3 (free or protein bound), TSH and other biomarkers using radioimmunoassay or liquid chromatography-tandem mass spectrometry (LC-MS/MS) technology (43). In particular, “third generation” TSH tests are now sufficiently sensitive to detect TSH levels of <0.01 mIU/L, for use in diagnosing subclinical hypothyroidism or hyperthyroidism (44). The availability of accurate tests for thyroid hormones facilitated the diagnosis of thyroid dysfunction and guided dose titration: indeed, the advent of accurate thyroid function tests revealed that many patients with hypothyroidism had been over treated, with a resulting reduction in the LT4 dose of half or more (10).

The application of LT4-based therapy for hypothyroidism continues to be refined. Regulators define LT4 as a “narrow therapeutic index” drug, meaning that even a small alteration in exposure to LT4 can result in clinically important biological consequences. This has led regulators to impose increasingly stringent criteria in recent years for the manufacture of LT4 tablets, with regard to the accuracy and reproducibility of their LT4 content and the stability of the active ingredient over time (45–47). This requirement has mandated re-engineering of existing LT4 tablets to meet these new quality standards (48, 49), which should support more accurate titration and maintenance treatment for people with hypothyroidism who require treatment with LT4.

These developments have established LT4 as the standard of care for the management of hypothyroidism. Hypothyroidism is a common condition affecting some 3–11% of local populations. It has been reported that LT4 is the second-most-used prescription drug by outpatients in the USA, with more than 20 million patients receiving almost 99 million prescriptions in 2020 (50).

## Looking ahead

### What about T3 replacement?

Combination treatment with LT4 and T3 (liothyronine) for people with hypothyroidism was common up to about 1970, as it was assumed that this was an obvious approach to mimicking natural thyroid function. The discovery of peripheral iodothyronine deiodinases reduced the perceived need for this approach, along with the observation that monotherapy with LT4 was sufficient for most people with hypothyroidism. In addition, clinical trials comparing LT4-T3 combinations with LT4 monotherapy produced variable results, with no clear advantage for the combination. A re-evaluation of those trials has identified methodological shortcomings, however, including recruitment of patients without clear T3 deficiency (or a deiodinase polymorphism that predisposes to peripheral T3 deficiency), differences in the extent of residual thyroid function at baseline, and a lack of appropriate thyroid dysfunction-specific instruments for recording changes in symptoms and patient-reported outcomes (51–54). In addition, the widely divergent plasma half-lives of available preparations of T4 (days) and T3 (hours) complicates their co-administration in a once-daily dose (as is the case for LT4 monotherapy). New and more appropriately designed trials, ideally using a T3 product with a longer half-life, will be needed to address these issues (2, 55).

Most cases of hypothyroidism can be controlled adequately using LT4 monotherapy, as described above. However, a minority of LT4-treated patients continue to report symptoms reminiscent of hypothyroidism despite having TSH controlled to within the reference range (56). Careful examination may reveal a hitherto undiscovered explanation for these symptoms in most, but not all, patients. Variations in the activity of deiodinases, in part due to LT4 treatment, may alter the relative availability of T4 and T3 in peripheral target tissues, which may underlie the persistence of hypothyroid symptoms in some patients (57, 58). Such observations have increased interest in the use of LT4-T3 combinations. Current European guidance supports a trial of this combination therapy for selected patients with symptoms of hypothyroidism that persist despite optimised LT4 treatment. Again, further clinical trials are required to quantify the benefits of this approach.

### Key outstanding research questions

Two important questions for the future of thyroid research have already been mentioned above, namely relate to whether genetic polymorphisms in deiodinases lead to clinically significant variations in the ability of LT4-based therapy to restore normal thyroid function in an individual with hypothyroidism, and the related but separate question of the role (if any) of T3 in the management of hypothyroidism. The associations between thyroid dysfunction and comorbid conditions requires further study: for example, low T3 is a common finding in patients with heart failure and may contribute to the pathophysiology of this disorder (59).

In addition, each patient may have their own unique “set point” for thyroid homeostasis, and it is possible that one or more thyroid hormones will lie outside their reference range without adverse consequences for thyroid homeostasis in that individual (60). Other authors have questioned the existence of these set points, however, and research continues to provide the optimum definition of euthyroidism, perhaps beyond the use of TSH as the primary biomarker (61). Finally, the management of subclinical hypothyroidism and the extent to which this condition is associated with severe adverse clinical outcomes provides a continuing research challenge (62). This is especially relevant to elderly people with mild elevations of TSH that likely result from a natural age-related process rather than genuine thyroid pathology (63).

## Conclusions

**Figure 2:**
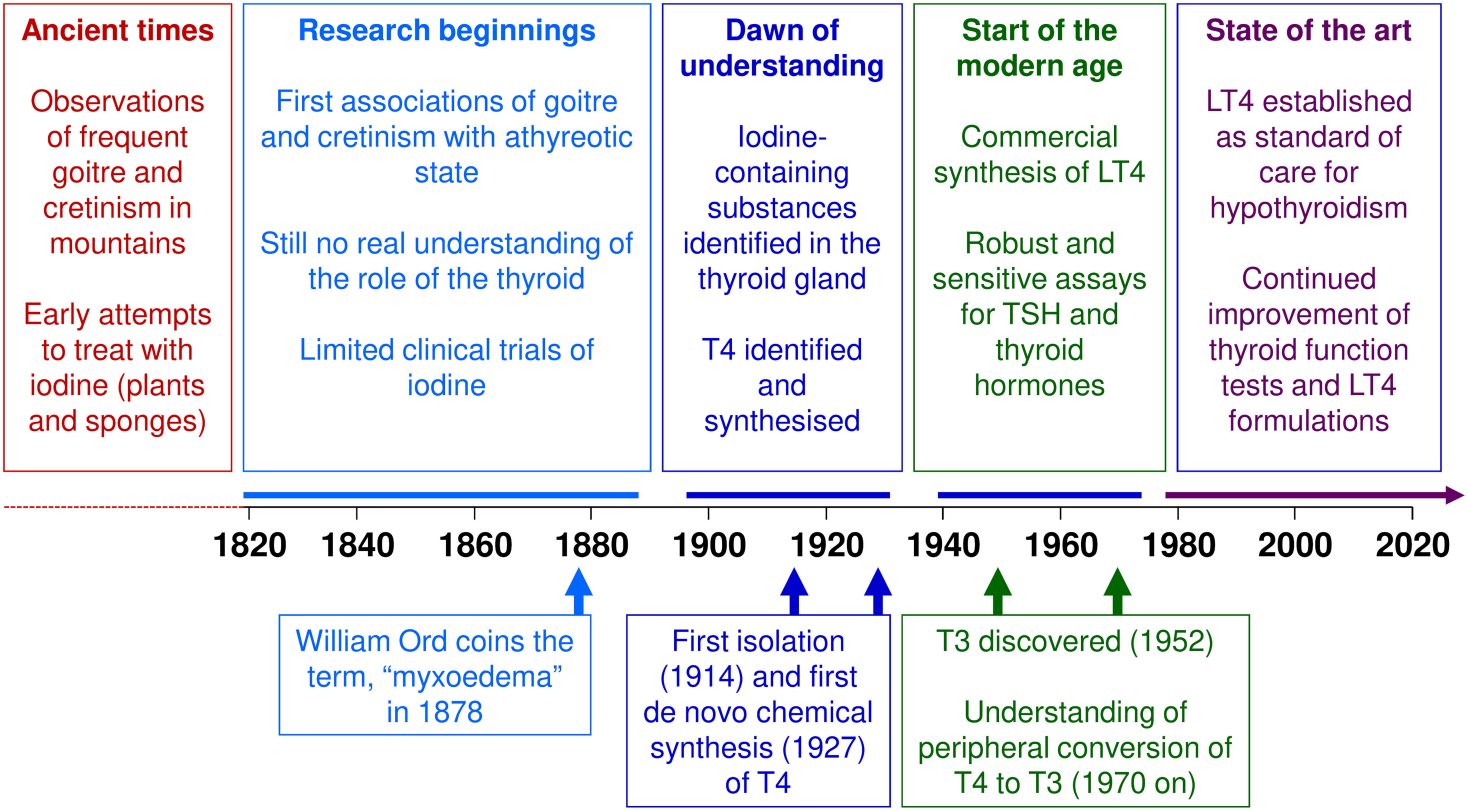
Key events in the history of levothyroxine (LT4). T3, triiodothyronine; T4, thyroxine; TSH, thyrotropin/thyroid stimulating hormone.

The devastating consequences of untreated thyroid dysfunction have been evident for centuries. Clinical research conducted over the last two centuries first associated goitre and cretinism with iodine status, and later with thyroid dysfunction (**Figure 2**). Research in the first half of the 20^th^ century laid the groundwork for our current understanding of thyroid hormones in health and disease that we have today, but it was not until the second half of that century that synthetic LT4 emerged as the mainstay of treatment for hypothyroidism. Today, LT4 monotherapy, titrated to normalise the circulating level of TSH, is the standard of care for the management of hypothyroidism. Future research will no doubt refine this management of thyroid disease.

## Author contributions

GJK is the senior author. Both authors contributed to the development of the article and approved the submitted version.

## Funding

Merck Healthcare KGaA, Darmstadt, Germany funded expedited review, colour figures and editorial support (see below).

## Acknowledgments

A medical writer (Dr Mike Gwilt, GT Communications) provided editorial assistance, funded by Merck healthcare KGaA (CrossRef Funder ID: 10.13039,100009945).

## Conflict of interest

The JGU Medical Center, Mainz, Germany receives research-associated funding from Merck Healthcare KGaA, Darmstadt, Germany. UG-H is an employee of Merck Healthcare KGaA.

The authors declare that the research was conducted in the absence of any commercial or financial relationships that could be construed as a potential conflict of interest.

## Publisher’s note

All claims expressed in this article are solely those of the authors and do not necessarily represent those of their affiliated organizations, or those of the publisher, the editors and the reviewers. Any product that may be evaluated in this article, or claim that may be made by its manufacturer, is not guaranteed or endorsed by the publisher.

## References

[1] Hoermann R Midgley JE Larisch R Dietrich JW . Homeostatic control of the thyroid-pituitary axis: Perspectives for diagnosis and treatment. Front Endocrinol (2015) 6:177. doi: 10.3389/fendo.2015.00177 PMC4653296 26635726

[2] Hennessey JV . Therapeutic actions of levothyroxine, in: ‘70 years of levothyroxine’ . Cham, Switzerland: Springer Nature Switzerland AG. Available at: https://link.springer.com/book/10.1007/978-3-030-63277-9 (Accessed September 2022).

[3] Okosieme O Gilbert J Abraham P Boelaert K Dayan C Gurnell M . Management of primary hypothyroidism: statement by the British thyroid association executive committee. Clin Endocrinol (2016) 84:799–808. doi: 10.1111/cen.12824 26010808

[4] National Institute for Health and Care Excellence . Clinical knowledge summaries. hypothyroidism. last revised in June 2018. Available at: https://cks.nice.org.uk/hypothyroidism (Accessed March 2019).

[5] Jonklaas J Bianco AC Bauer AJ Burman KD Cappola AR Celi FS . Guidelines for the treatment of hypothyroidism: prepared by the American thyroid association task force on thyroid hormone replacement. Thyroid (2014) 24:1670–751. doi: 10.1089/thy.2014.0028 PMC4267409 25266247

[6] Sheehan MT . Biochemical testing of the thyroid: TSH is the best and, oftentimes, only test needed - a review for primary care. Clin Med Res (2016) 14:83–92. doi: 10.3121/cmr.2016.1309 27231117 PMC5321289

[7] Pearce SH Brabant G Duntas LH Monzani F Peeters RP Razvi S . 2013 ETA Guideline: management of subclinical hypothyroidism. Eur Thyroid J (2013) 2:215–28. doi: 10.1159/000356507 PMC3923601 24783053

[8] Chaker L Razvi S Bensenor IM Azizi F Pearce EN Peeters RP . Hypothyroidism. Nat Rev Dis Primers (2022) 8:30. doi: 10.1038/s41572-022-00357-7 35589725

[9] Lindholm J Laurberg P . Hypothyroidism and thyroid substitution: historical aspects. J Thyroid Res (2011), 2011:809341. doi: 10.4061/2011/809341 21760981 PMC3134382

[10] McAninch EA Bianco AC . The history and future of treatment of hypothyroidism. Ann Intern Med (2016) 164:50–6. doi: 10.7326/M15-1799 PMC4980994 26747302

[11] Slater S . The discovery of thyroid replacement therapy. part 1: In the beginning. J R Soc Med (2011) 104:15–8. doi: 10.1258/jrsm.2010.10k050 PMC3014555 21205773

[12] Sterpetti AV . How the art in Rome represented personages with goitre. Eur J Intern Med (2016) 32:e28–9. doi: 10.1016/j.ejim.2016.03.023 27083552

[13] Trinca F Riva MA . The representation of a goiter by renaissance painter moretto da brescia. J Endocrinol Invest (2019) 42:1133–4. doi: 10.1007/s40618-019-01023-8 30778906

[14] Sterpetti AV Fiori E De Cesare A . Goiter in the art of renaissance Europe. Am J Med (2016) 129:892–5. doi: 10.1016/j.amjmed.2016.04.015 27154774

[15] Vescia FG Basso L . Goiters in the renaissance. Vesalius (1997) 3:23–32. 11619418

[16] Ferriss JB . The many reasons why goiter is seen in old paintings. Thyroid (2008) 18:387–93. doi: 10.1089/thy.2007.0301 18399764

[17] Niazi AK Kalra S Irfan A Islam A . Thyroidology over the ages. Indian J Endocrinol Metab (2011) 15(Suppl 2):S121–6. doi: 10.4103/2230-8210.83347 PMC3169859 21966648

[18] Alexander JL Astill PH Emerson JW Evans SM Harcus AW Holden JSc . Derbyshire Neck. thyroid abnormalities in the Derbyshire peak district. Lancet (1966) 2:959–61. doi: 10.1016/S0140-6736(66)90556-3 4162384

[19] Curling TB . Two cases of absence of the thyroid body, and symmetrical swellings of fat tissue at the sides of the neck, connected with defective cerebral development. Med ChirTrans (1850) 33:303–6. doi: 10.1177/095952875003300123 PMC2104224 20895941

[20] Fagge CH . On sporadic cretinism, occurring in England. Med Chir Trans (1871) 54:55–169. doi: 10.1177/095952877105400108 PMC2150485 20896365

[21] Pearce JM . Myxoedema and sir William withey gull (1816-1890). J Neurol Neurosurg Psychiatry (2006) 77:639. doi: 10.1136/jnnp.2005.082198 16614024 PMC2117466

[22] Ord WM . On myxœdema, a term proposed to be applied to an essential condition in the "Cretinoid" affection occasionally observed in middle-aged women. Med Chir Trans (1878) 61:57–78. doi: 10.1177/095952877806100107 PMC2150242 20896517

[23] Hirsch A . Handbook of geographical and historical pathology: Volume II. (London: New Sydenham Society) (1885).

[24] Horsley V . On the function of the thyroid gland. Proc R Soc Lond (1885) 38:5–7. doi: 10.1098/rspl.1884.0054

[25] Schiff M . Résumé d’une nouvelle série d’expériences sur les effets de l’ablation des corps thyroîdes. In: Revue médicale de la suisse romande (Geneve: Imprimerie Charles Schuchardt), vol. 4. (1884). p. 425–45.

[26] Kocher T . Ueber kropf exstirpation und ihre folgen. Archiv Klinische Chirurgie (1883) 29:254–335.

[27] Zimmermann MB . Research on iodine deficiency and goiter in the 19th and early 20th centuries. J Nutr (2008) 138:2060–3. doi: 10.1093/jn/138.11.2060 18936198

[28] Coindet JF . Nouvelles recherches sur les effets de l'iode et sur les précautions à suivre dans le traitement du goître par ce nouveau remède. Ann Chim Phys (1821) 16(Ser. 2):345–56.

[29] Chatin A . Recherches sur l'iode des eaux douces) de la présence de ce corps dans les plantes at les animaux terrestes. In: Comptes rendus hebdomadaires des séances de l'Academie des sciences (Paris: Gauthier-Villars), vol. 31. (1851). p. 280–3.

[30] Doyle L . Myxoedema: Some early reports and contributions by British authors, 1873-1898. J R Soc Med (1991) 84:103–6. doi: 10.1177/014107689108400216 PMC1293099 1999807

[31] Murray GR . The life-history of the first case of myxoedema treated by thyroid extract. Br Med J (1920) 3089:359–60. doi: 10.1136/bmj.1.3089.359 PMC2337775 20769820

[32] Beadles CF . The treatment of myxœdema and cretinism, being a review of the treatment of these diseases with the thyroid gland, with a table of 100 published cases. J Ment Sci (1893) 39:509–36. doi: 10.1192/bjp.39.167.509

[33] Baumann E . Ueber das normale vorkommen von jod im thierkörper. Hoppe-Seyler's Z fur Physiologische Chemie (1895) 21:319–30. doi: 10.1515/bchm2.1896.21.4.319

[34] Ahmed AM Ahmed NH . History of disorders of thyroid dysfunction. East Mediterr Health J (2005) 11:459–69. doi: 10.26719/2005.11.3.459 16602467

[35] Kendall EC . The isolation in crystalline form of the compound containing iodin, which occurs in the thyroid: Its chemical nature and physiologic activity. J Am Med Ass (1915) 64:2042–3. doi: 10.1001/jama.1915.02570510018005 6352971

[36] Bramwell B . The thyroid treatment of myxoedema and sporadic cretinism, with notes of twenty-three cases of myxoedema and five cases of sporadic cretinism, treated by thyroid extract. Edinburgh Hosp Rep (1895) 3:116–249.

[37] Harington CR Barger G . Chemistry of thyroxine. III: Constitution and synthesis of thyroxine. Biochem J (1927) 21:169–83. doi: 10.1042/bj0210169 PMC1251886 16743801

[38] Chalmers JR Dickson GT Elks J Hems BA . The synthesis of thyroxine and related substances. part v. a synthesis of l-thyroxine from l-tyrosine. J Chem Soc (1949) 3424–38.

[39] Mateo RCI Hennessey JV . Thyroxine and treatment of hypothyroidism: seven decades of experience. Endocrine (2019) 66:10–7. doi: 10.1007/s12020-019-02006-8 PMC6794242 31321670

[40] Braverman LE Ingbar SH Sterling K . Conversion of thyroxine (T4) to triiodothyronine (T3) in 42. athyreotic human subjects. J Clin Invest (1970) 49:855–64. doi: 10.1172/JCI106304 PMC535757 4986007

[41] Steegborn C Schweizer U . Structure and mechanism of iodothyronine deiodinases - what we know, what we don't know, and what would be nice to know. Exp Clin Endocrinol Diabetes (2020) 128:375–8. doi: 10.1055/a-1022-9916 31698481

[42] Tata JR . Rosalind Pitt-rivers and the discovery of T3. Trends Biochem Sci (1990) 15:282–4. doi: 10.1016/0968-0004(90)90055-g 2200172

[43] Spencer CA . Assay of thyroid hormones and related substances. Available at: https://www.ncbi.nlm.nih.gov/books/NBK279113/ (Accessed March 2020).

[44] Kuy JM . The evolution of thyroid function tests. J Endocrinol Metab Diabetes S Afr (2015) 20:11–6. doi: 10.1080/16089677.2015.1056468

[45] Medicines and health regulatory authority. levothyroxine tablet products: a review of clinical & quality considerations (2013). Available at: http://webarchive.nationalarchives.gov.uk/20141205150130/http:/www.mhra.gov.uk/home/groups/pl-p/documents/drugsafetymessage/con222566.pdf (Accessed September 2018).

[46] Agence française de sécrurité sanitaire des produits de santé, in: Commission nationale de pharmacovigilance. compte rendu de la réunion du mardi (2012). Available at: https://ansm.sante.fr/var/ansm_site/storage/original/application/4e4d2a70e5dddfb150fe87360d6b13dd.pdf (Accessed September 2012).

[47] The united states pharmacopeial convention 2009 current USP monograph of levothyroxine sodium tablets (published in revision bulletin, official February 1, 2010). Available at: https://www.uspnf.com/sites/default/files/usp_pdf/EN/USPNF/levothyroxineSodiumTablets.pdf (Accessed September 2010).

[48] Gottwald-Hostalek U Uhl W Wolna P Kahaly GJ . New levothyroxine formulation meeting 95-105% specification over the whole shelf-life: results from two pharmacokinetic trials. Curr Med Res Opin (2017) 33:169–74. doi: 10.1186/s12902-019-0365-4 27718637

[49] Lipp HP Hostalek U . A new formulation of levothyroxine engineered to meet new specification standards. Curr Med Res Opin (2019) 35:147–50. doi: 10.1159/000339444 30406687

[50] ClinCalc DrugStats Database . Available at: https://clincalc.com/DrugStats/Default.aspx (Accessed September 2022).

[51] Hennessey JV Espaillat R . Current evidence for the treatment of hypothyroidism with levothyroxine/levotriiodothyronine combination therapy versus levothyroxine monotherapy. Int J Clin Pract (2018) 72:e13062. doi: 10.1080/03007995.2021.1984219 PMC5873391 29381251

[52] Midgley JEM Toft AD Larisch R Dietrich JW Hoermann R . Time for a reassessment of the treatment of hypothyroidism. BMC Endocr Disord (2019) 19:37. doi: 10.1530/EC-20-0205 30999905 PMC6471951

[53] Wiersinga WM Duntas L Fadeyev V Nygaard B Vanderpump MP . 2012 ETA Guidelines: The use of l-T4 + l-T3 in the treatment of hypothyroidism. Eur Thyroid J (2012) 1:55–71. doi: 10.1159/000339444 24782999 PMC3821467

[54] DiStefano J 3rd Jonklaas J . Predicting optimal combination LT4 + LT3 therapy for hypothyroidism based on residual thyroid function. Front Endocrinol (2019) 10:746. doi: 10.3389/fendo.2019.00746 PMC6873785 31803137

[55] Gottwald-Hostalek U Kahaly GJ . Triiodothyronine alongside levothyroxine in the management of hypothyroidism? Curr Med Res Opin (2021) 37:2099–106. doi: 10.1530/EJE-12-0627 34553643

[56] Razvi S Mrabeti S Luster M . Managing symptoms in hypothyroid patients on adequate levothyroxine: a narrative review. Endocr Connect (2020) 9:R241–50. doi: 10.1210/jc.2003-031641 PMC7774765 33112818

[57] Bianco AC . Cracking the code for thyroid hormone signaling. Trans Am Clin Climatol Assoc (2013) 124:26–35. 23874007 PMC3715916

[58] Peeters RP Visser TJ . Metabolism of thyroid hormone. Available at: https://www.ncbi.nlm.nih.gov/books/NBK285545 (Accessed February 2020).

[59] Biondi B . Mechanisms in endocrinology: Heart failure and thyroid dysfunction. Eur J Endocrinol (2012) 167:609–18. doi: 10.1210/jc.2003-031641 22956554

[60] Hansen PS Brix TH Sørensen TI Kyvik KO Hegedüs L . Major genetic influence on the regulation of the pituitary-thyroid axis: a study of healthy Danish twins. J Clin Endocrinol Metab (2004) 89:1181–87. doi: 10.1210/jc.2003-031641 15001606

[61] Fitzgerald SP Falhammar H . Redefinition of successful treatment of patients with hypothyroidism. is TSH the best biomarker of euthyroidism? Front Endocrinol (2022) 13:920854. doi: 10.1136/bmj.l2006 PMC9243528 35784560

[62] Bekkering G Agoritsas T Lytvyn L Heen AF Feller M Moutzouri E . Thyroid hormones treatment for subclinical hypothyroidism: a clinical practice guideline. BMJ (2019) 365:l2006. doi: 10.1016/S0021-9258(18)87324-0 31088853

[63] Calsolaro V Niccolai F Pasqualetti G Calabrese AM Polini A Okoye C . Overt and subclinical hypothyroidism in the elderly: When to treat? Front Endocrinol (2019) 10:177. doi: 10.1055/a-1022-9916 PMC6438852 30967841

